# Retinal telangiectasia-like lesions in a 15-year-old female with Hereditary hemorrhagic telangiectasia – a case report

**DOI:** 10.1186/s12886-022-02658-7

**Published:** 2022-11-07

**Authors:** Ardiana Ala, Torben Lykke Sørensen, Caroline Schmidt Laugesen

**Affiliations:** Department of Ophthalmology, Zealand University Hospital, Vestermarksvej 23, 4000 Roskilde, Denmark

**Keywords:** Hereditary hemorrhagic telangiectasia, Rendu-Osler-Weber, Retinal telangiectasia, Retinal abnormalities, Case report

## Abstract

**Background:**

Hereditary hemorrhagic telangiectasia (HHT), also known as Rendu-Osler-Weber syndrome is a bleeding disorder that can affect all parts of the body including the eyes. Different ocular abnormalities have been described in relation to HHT, but the pathogenesis of retinal involvement is still unknown. A few cases have described chorioretinal abnormalities primarily occurring in elderly patients. In this study, we present a unique case of a young female with known HHT and a series of retinal fundus images including optical coherence tomography (OCT) and optical coherence tomography angiography (OCTA) with macular telangiectasia-like lesions.

**Case presentation:**

A young female genetically diagnosed with hereditary hemorrhagic telangiectasia (HHT), is regularly attending retinal screening since she is diagnosed with Type 1 diabetes. At one visit, abnormal retinal telangiectasia-like lesions in the macula, are observed. These abnormalities are monitored over an extended period of time with fundus imaging, and further investigated with OCT and OCTA. The patient has no visual complaints at any time and best-corrected visual acuity is 20/20 Snellen equivalent in both eyes.

**Conclusions:**

To the best of our knowledge, this is the first case to describe the occurrence of telangiectasia-like lesions in macula with secondary choriocapillaris atrophy in a patient diagnosed with HHT in such a young age.

**Supplementary Information:**

The online version contains supplementary material available at 10.1186/s12886-022-02658-7.

## Background

Hereditary hemorrhagic telangiectasia (HHT) also known as Rendu-Osler-Weber syndrome, is an autosomal, dominantly inherited bleeding disorder [1]. HHT is characterized by the presence of vascular malformations. Nose bleeding is the most common symptom, but bleeding can also be cutaneous, gastrointestinal, or ocular. Bleeding in HHT is secondary to malformed blood vessels and increased fragility rather than the risk of bleeding due to plasma coagulation factors or platelet dysfunction associated with surgical procedures [2]. HHT is diagnosed clinically and/or by genetic testing. The clinical diagnostic is based on four criteria (at least three of these have to be present), commonly referred to as Curaҫao criteria, which are the following: (i) recurrent spontaneous epistaxis, (ii) multiple telangiectasias, (iii) visceral vascular malformations especially in lungs, liver, and brain or (iiii) a compatible family history [1].

Ocular manifestations in HHT include conjunctival, retinal, and choroidal telangiectasias [3–5]. The most frequent are the conjunctival appearances of telangiectasias and hemorrhagic epiphora [3, 4]. Previously reported intraocular abnormalities include telangiectasias, neovascularization, and dilated and tortuous retinal vessels [5]. The prevalence of these abnormalities vary among the reported papers [5].

Here we present a unique case of a young female with known HHT type 2 and a series of retinal fundus images including OCT and OCTA followed over several years with chorioretinal abnormalities in both eyes.

## Case presentation

A 15-year-old female with diabetes type 1 is followed in an outpatient clinic, as part of the Ophthalmology Department at Zealand University Hospital Roskilde, Denmark.

She has a family history of HHT and by the age of 12, she is diagnosed with HHT (mutation ACVRL c1232G > A). Her primary complaint related to HHT is recurrent nose bleeding. A thorough clinical examination concerning HHT has only shown a few telangiectasias on her fingers and in the nose.

**Fig. 1:**
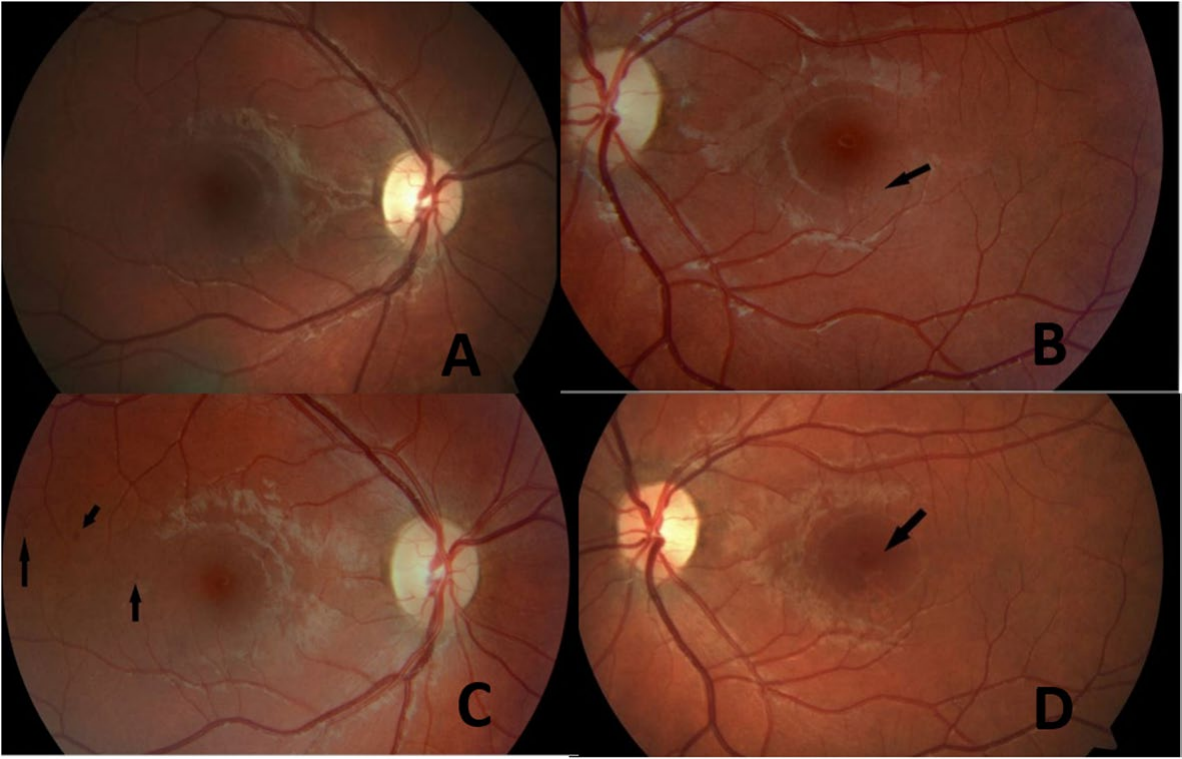
**A**. Fundus image of the right eye in 2013. **B** Fundus image of the left eye in 2016, showing several subtle pigmentary changes in the parafoveal area, indicated by arrow. **C** Fundus image of the right eye in 2018, showing three telangiectasias in the temporal area indicated by arrows. **D** Fundus image of the left eye in 2019, showing the telangiectasia-like lesion in fovea. The pigmentary lesions in the parafoveal area are unchanged

She started the diabetic screening program from the age of 12 and has since then been followed continuously over the years. The first eye-screening visit took place in 2013. No visual complaints nor any retinal abnormalities were observed (Fig. 1 A). In 2016, fundus images of the left eye showed signs of pigmentary changes located in the area around the fovea (Fig. 1 B). There were no changes in the right eye. Two years later, the lesions were more pigmented, indicating that the changes probably are located in the retinal pigment epithelium layer and thus not related to diabetic retinopathy. Simultaneously, the right eye had developed three small retinal abnormalities imitating macular telangiectasia-like lesions (Fig. 1 C).

Approximately 6 months later, new telangiectatic changes in the left eye occurred (Fig. 1 D). Since OCT was not available in the outpatient clinic the patient was referred to the Eye Department in Zealand University Hospital, Roskilde for further examination. Fundoscopy, OCT and OCTA scans were collected on the Spectralis HRA + OCT machine (spectral domain optical coherence tomography (OCT) (SPECTRALIS® HRA + OCT, Heidelberg Engineering, Heidelberg Germany)).

**Fig. 2:**
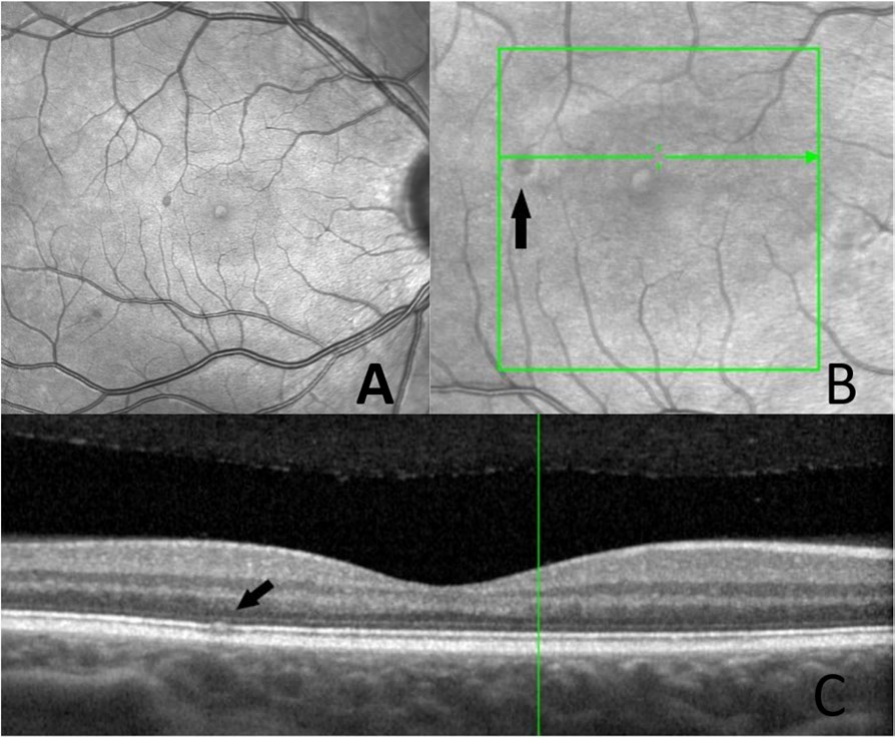
**A** Fundus image of the right eye with the parafoveal telangiectasia. **B** and **C**. OCT of the right eye showing a shadow like modification in the macula, corresponding to one of the three changes seen by fundus imaging

**Fig. 3:**
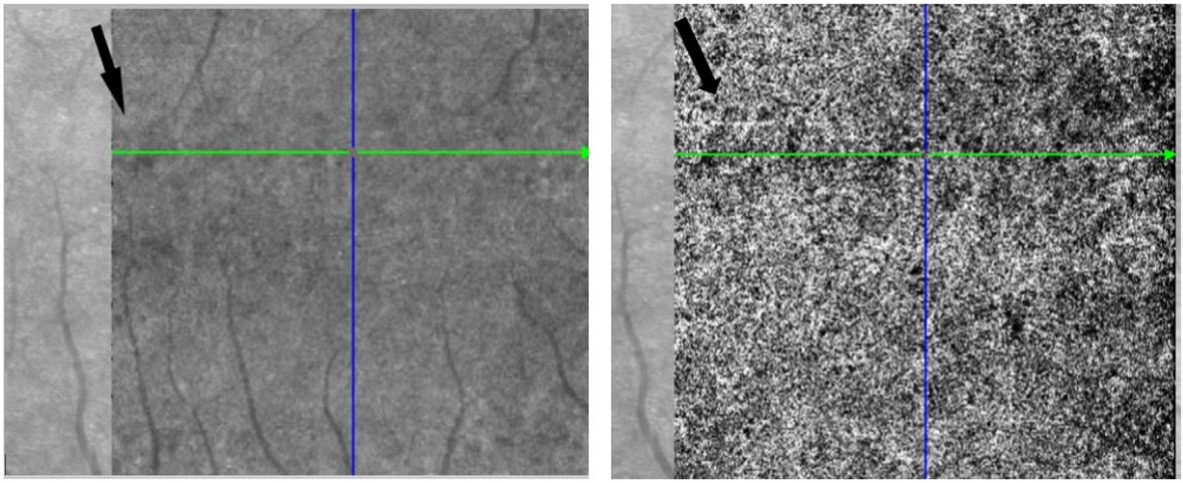
OCTA of the right eye showing the choriocapillaris layer. The arrow marking the change, corresponding the telangiectasia-like lesion

**Fig. 4:**
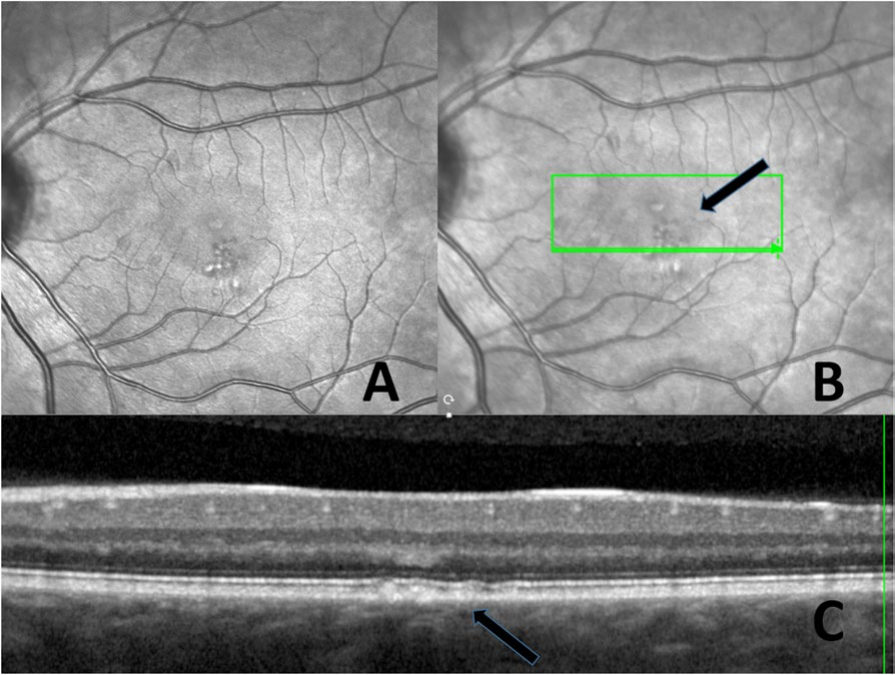
**A** Infrared image of the left eye visualizing the atrophies and just above these parafoveal telangiectasias. **B** OCT of the left eye with an arrow indicating the areas of pigmentary changes. **C** OCT showing the lesions in the fovea located in the pigment epithelial layer

**Fig. 5:**
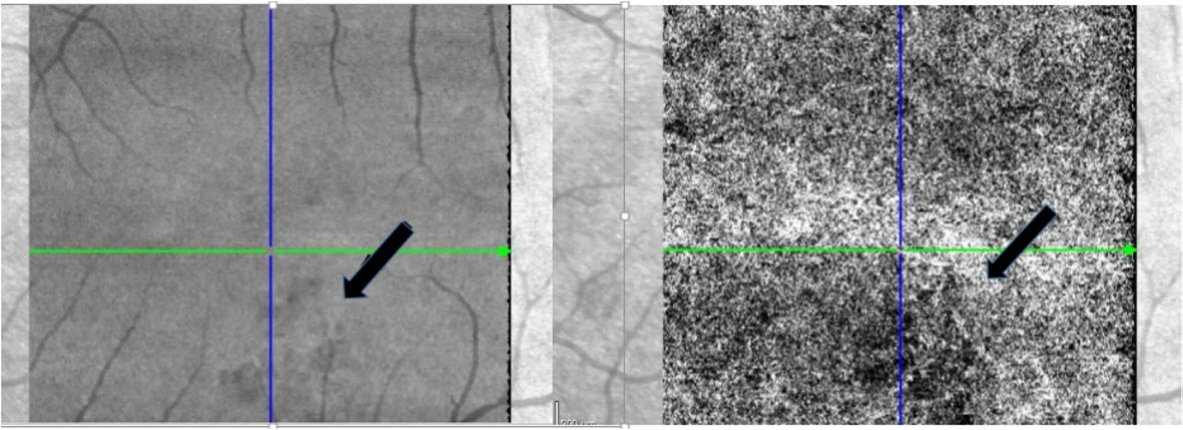
OCTA of the left eye showing the choriocapillaris layer corresponding to the area of pigmentary changes

The OCT demonstrated that the lesions are located close to the RPE (retinal pigment epithelial layer) (Fig. 2). The changes are more obvious using the infrared image modality (Fig. 2. A). OCTA showed that the changes seen in the right eye, are located in the choriocapillaris layer (Fig. 3) corresponding to the same place the teleangiectasia-like lesions are seen on earlier fundus images. The changes in the left eye (Fig. 4), are also located in the choriocapillaris expanding towards the retinal pigment epithelial layer, corresponding to the pigmentary changes in the parafoveal area (Fig. 5).

At all screening visits, the patient had no visual complaints and had never undergone ocular surgery, laser treatment, or ocular trauma. Best-corrected visual acuity was 90 ETDRS (Early Treatment Diabetic Retinopathy Study) letters on both eyes and there has not been any sign of DR.

## Discussion and conclusions

Several cases of ocular involvement in patients with HHT have been described, though it remains unclear whether these changes are permanently, and which effect they have on visual acuity.

In this case, we present a young female with a continuous follow-up extending over a period of nine years where the lesions, probably due to HHT type 2, evolve over time. Her best-corrected visual acuity is unaffected at all time and there are no visual complaints despite the objective findings by fundus imaging, infrared images, OCT and OCTA verifying changes in the choriocapillaris and retinal pigment epithelial layers.

Whether the genetic variant influences on ocular changes is unknown. According to a study by Ines Gomez-Acebo e al. [4] ocular lesions are mainly associated with HHT 1 subtype. The patient in this case has the HHT 2 subtype, which does not correspond to the findings of Ines Gomez-Acebo e al. [4].

Earlier studies have found chorioretinal lesions in the eyes of elderly patients. To our knowledge chorioretinal changes have not been reported before in young patients. Sindhar et al. found no lesions in patients younger than the mean age (52 years), suggesting that the lesions may develop later in life [5].

Compared to conjunctival telangiectasias, chorioretinal findings are rare in patients with HHT. Although a study by Rinaldi et al. found 3 out of 8 patients with intraocular lesions described as widened and well-defined areas of choriocapillaris atrophy, and to a less extent involvement of the retinal pigment epithelium [6]. This could be the same type of lesion, seen in this case. In the study by Rinaldi et al. the average age of the patients with intraocular lesions is 60 years (range 57–62) and a visual acuity of (0.5—0.8). It has been suggested that an extension of the choriocapillary changes may lead to visual impairment [6].

Another case with similar changes reports retinal pigment modifications overlying choroidal ectatic vessels [7]. Here the authors suggest that the altered choroidal vessels could be the cause of changes in the retinal pigment epithelium, possibly owing to micro-excudation from the choriocapillaris layer.

Also, a case report by Mennel et al. [8] in 2005 reveals parafoveal telangiectasia in both eyes seen by fluorescein angiography in a 76- year old woman known with HHT. The authors conclude that a choroidal neovascularization (CNV) occurred secondly to the parafoveal telangiectasias in one eye. The CNV was treated with photodynamic therapy.

A study by Sindhar et al. [5], who examined eighteen patients having HHT with fluorescein angiography, found 83% of the patients with retinal alterations. The occurrence of ocular findings in these patients appears to be more frequent and as they suggest, the alterations might be over-looked with fundus photography.

A recent article by Abdolrahimzadeh et al. [9] discusses the different examination modalities of patients and that this may be a factor in the variation between the different frequencies of the retinal abnormalities found amongst studies. This aspect is also supported by Sindhar et al. [5] as they used both fluorescein angiography and OCTA in their study of HHT patients. They also found the highest occurrence of intraocular malformations (78%) and that is probably due to the fact of the different examination modalities [5, 9]. Ocular involvement in HHT patients is more common than assumed. Even though intraocular involvement is relatively rare. Conjunctival lesions seem to be more harmless, while the consequences of the intraocular lesions are unknown.

Our case is unique primarily because of the young age of the patient and because of the time spectrum. In this case, lesions develop over a long period in both eyes.

It is not possible to conclude from this case whether there is an indication for screening of this patient group. The disease is rare, the conjunctival affections are harmless and the consequences of the more uncommon retinal lesions are unknown.

## Supplementary Information

**Additional file 1.**

## Data Availability

Not applicable.

## Abbreviations

HHT: Hereditary hemorrhagic telangiectasia
OCT: Optical coherence tomography
OCTA: Optical coherence tomography angiography
DR: Diabetic retinopathy
CNV: Choroidal neovascularization
ETDRS: Early Treatment Diabetic Retinopathy Study
ACVRL: Activin A receptor like kinase type 1
